# Clay-Based Pharmaceutical Formulations and Drug Delivery Systems

**DOI:** 10.3390/pharmaceutics12121142

**Published:** 2020-11-25

**Authors:** Fátima García-Villén, César Viseras

**Affiliations:** 1 Department of Pharmacy and Pharmaceutical Technology, Faculty of Pharmacy, University of Granada, Campus of Cartuja, 18071 Granada, Spain; fgarvillen@ugr.es; 2 Andalusian Institute of Earth Sciences, CSIC-UGR, Avenida de las Palmeras 4, 18100 Armilla, Granada, Spain

The use of minerals as ingredients in health care products is a classical and active pharmaceutical subject. Clays and also zeolites and other silica-based mesoporous inorganic ingredients have been traditionally used as pharmaceutical and cosmetic ingredients. These inorganic ingredients should meet the quality and safety standards to be used as ingredients in health care products. Recently, new and advanced applications of these materials have been proposed, including the design of modified drug delivery systems and other advanced applications (such as wound healing formulations and tissue engineering scaffolds).

This Special Issue highlights the most relevant and recent advances in clay-based formulations and clay-based drug delivery systems. Natural, modified, and synthetic clays with prospects in nanomedicine and pharmaceutics were considered.

The publication of Maestrelli et al. [1] used a combination of cyclodextrins and nanoclays to improve the biopharmaceutical profile of hydrochlorothiazide, characterized by low solubility and permeability. Once the best cyclodextrin (RAMEB) and clay (sepiolite) were selected, the co-evaporation technique was used to prepare a ternary system with an optimal drug:carrier ratio. The combined presence of RAMEB and sepiolite gave rise to a synergistic improvement in the drug dissolution properties, resulting in an approximately 12-fold increase in the hydrochlorothiazide solubility compared with the drug alone. Subsequently, the ternary system was formulated as tablets and a full technological characterization was performed. The results clearly revealed a better drug dissolution performance than the commercial hydrochlorothiazide reference tablet (Esidrex^®^).

The publication by Borrego-Sánchez et al. [2] studied the interaction between praziquantel, the drug indicated for schistosomiasis disease, and two clays (sepiolite and montmorillonite). Praziquantel has a very low aqueous solubility, requiring high oral doses, which usually lead to side effects, therapeutic noncompliance, and the appearance of resistant forms of the parasite. The drug was dissolved in organic solvents (ethanol, acetonitrile, and dichloromethane) and encapsulated in nanometric channels of sepiolite or between the layers of montmorillonite. The results showed that the interaction of the drug with both clay minerals produced a loss of praziquantel crystallinity, as demonstrated by different techniques. This led to a significant increase in the dissolution rate of praziquantel in simulated gastrointestinal tract media, except for the praziquantel–montmorillonite product prepared in dichloromethane, which presented a controlled release in acid medium. Moreover, the drug–clay interaction products prepared in ethanol were subjected to in vitro cytotoxicity and cell cycle studies. The interaction product with sepiolite was biocompatible with the HTC116 line cells, and it did not produce alterations in the cell cycle. However, interaction products with montmorillonite did not produce cell death, but they altered the cell cycle at the highest concentration tested (20–100 µM). In conclusion, drug–clay interaction products, specifically with sepiolite, showed very promising results, since they accelerated praziquantel oral release.

Inorganic hydrogels formulated with spring waters and clay minerals are topically applied to treat musculoskeletal disorders and skin affections. From a pharmaceutical quality perspective, the safety limits of elemental impurities in clay-based hydrogels were studied by García-Villén et al. [3]. Their results showed that the release of a particular element not only depends on its concentration, but also on its position in the hydrogel network, concluding that hydrogels prepared with sepiolite, palygorskite, and local spring water could be topically applied without major intoxication risks. The wound healing properties of these clay-based hydrogels were addressed using Confocal Laser Scanning Microscopy to study the morphology of fibroblasts during the wound healing process. The studied clay-based hydrogels promoted in vitro fibroblast motility and, therefore, accelerated wound healing (García-Villén et al. [4]). The underlying mechanism of action for skin disorders of these formulations is usually ascribed to the chemical composition of the formulation. García-Villén et al. [5] assessed the composition and in vitro release of elements with potential wound healing effects from hydrogels prepared with two nanoclays and natural spring water. In vitro Franz cell studies were used and the element concentration was measured by the Inductively Coupled Plasma technique. Biocompatibility studies were used to evaluate the potential toxicity of the formulation against fibroblasts. The studied hydrogels released elements with known therapeutic potential in wound healing. The released ratios of some elements, such as Mg:Ca or Zn:Ca, played a significant role in the final therapeutic activity of the formulation. In particular, the proliferative activity of fibroblasts was ascribed to the release of Mn and the Zn:Ca ratio.

Pagano et al. [6] intercalated ketoprofen into a lamellar anionic clay ZnAl-hydrotalcite (ZnAl-HTlc), improving the stability to UV rays and the water solubility of the drug. The hybrid was then formulated in auto-adhesive patches for local pain treatment. The patches were prepared by a casting method, starting from a hydrogel based on the biocompatible and bioadhesive polymer NaCMC (Sodium carboxymethycellulose) and glycerol as a plasticizing agent. The addition of ZnAl-KET in the patch composition caused an improvement in the mechanical properties of the formulation. Moreover, a sustained and complete drug release was obtained within 8 h. This allowed reducing the frequency of anti-inflammatory posology compared to the conventional formulations.

Cirri et al. [7] designed fast-dissolving glyburide tablets based on a liquisolid approach using mesoporous clay (Neusilin^®^US2) or silica (Aeroperl^®^300) and dimethylacetamide or 2-pyrrolidone as drug solvents, without using the coating materials that are necessary in conventional systems. The resultant liquisolid tablets provided a marked drug dissolution increase, reaching 98% of dissolved drug after 60 min, compared to the 40% and 50% obtained from a reference tablet containing the plain drug and a commercial tablet. The improved glyburide dissolution was attributed to its increased wetting properties and surface area, due to its amorphization/solubilization within the liquisolid matrix, as confirmed by DSC and PXRD studies. Mesoporous clay and silica, owing to their excellent adsorbent, flow, and compressibility properties, avoided the use of coating materials while considerably improving the liquid-loading capacity, reducing the amount of carrier necessary to obtain freely flowing powders. Neusilin^®^US2 showed a superior performance with respect to Aeroperl^®^300 regarding the tablet’s technological properties.

Sandri et al. [8] designed and developed electrospun scaffolds, entirely based on biopolymers, loaded with montmorillonite or halloysite and intended for skin reparation and regeneration. The scaffolds were manufactured by means of electrospinning and were characterized for their chemico-physical and preclinical properties. The scaffolds proved to possess the capability to enhance fibroblast attachment and proliferation with negligible proinflammatory activity. The capability to facilitate the cell adhesion is probably due to their unique 3D structure, which assists in cell homing and would facilitate wound healing in vivo. Faccendini et al. [9] developed chitosan/glycosaminoglycan-based scaffolds loaded with norfloxacin (free or in montmorillonite hybrids). All the scaffolds were proven to be degraded via lysozyme (this should ensure scaffold resorption), and this sustained the drug release (from 50% to 100% in 3 days, depending on system composition), especially when the drug was loaded in the scaffolds as a clay-based nanocomposite. Moreover, the scaffolds were able to decrease the bioburden at least 100-fold, proving that drug loading in the scaffolds did not impair the antimicrobial activity of norfloxacin. Chondroitin sulfate and montmorillonite in the scaffolds are proven to possess a synergistic performance, enhancing the fibroblast proliferation without impairing norfloxacin’s antimicrobial properties. The scaffold based on chondroitin sulfate, containing 1% norfloxacin in the nanocomposite, demonstrated an adequate stiffness to sustain fibroblast proliferation and the capability to sustain antimicrobial properties to prevent/treat non-healing wound infections during the healing process.

The publication by Luo et al. [10] focused on halloysite nanotubes (HNTs) functionalized with folic acid to selectively target cancer cells and a fluorochrome to visualize the nanoparticle. The functionalized HNTs were loaded with methotrexate. and the cell viability, proliferation, and uptake efficiency in colon cancer, osteosarcoma, and a pre-osteoblast cell line (MC3T3-E1) were evaluated. The functionalized HNTs showed a high methotrexate loading efficiency and a prolonged release. Moreover, non-cancerous cells were unaffected after exposure to the formulation. Consequently, the nanoparticle designed was demonstrated to exclusively target cancer cells, which consequently reduces the methotrexate side-effects caused by the off-targeting of anti-cancer drugs.

This Special Issue evidences the high potential and versatility of clay minerals in pharmaceutics. Despite being ingredients whose use dates back to ancient times, they continue to play a crucial role in the present, both as excipients and actives in a wide variety of dosage forms and novel technological strategies, such as tissue engineering and targeted cancer treatments.
